# Correlations of gait kinematics and cognitive skills in Parkinson disease

**DOI:** 10.1371/journal.pone.0317389

**Published:** 2025-06-13

**Authors:** Joshua J. Lander, Matthew F. Moran, Hannah R. Alexanian

**Affiliations:** 1 Department of Physical Therapy and Human Movement Science, Sacred Heart University, Fairfield, Connecticut, United States of America; 2 Lander Sport and Health Sciences, Westport, Connecticut, United States of America; Rutgers University - Newark, UNITED STATES OF AMERICA

## Abstract

Cognitive impairments and gait disturbances are often concurrent in Parkinson disease (PD), but the extent to which specific cognitive deficits relate to gait abnormalities remains unclear, especially in early-stage PD. To address this gap, we conducted an observational correlational study to determine if cognitive performance in distinct domains is associated with gait kinematics in PD. This study included a cohort of 19 individuals with early-stage PD who underwent gait analysis with a three-dimensional marker-less motion capture system (Theia Markerless Inc., Kingston, ON, CA) during three conditions: single-task walking, cognitive dual-task walking, and the Timed Up and Go (TUG) test. Cognitive abilities were assessed using computerized tests (Creyos, Toronto, ON, CA) of working memory (WM), response inhibition (RI), and mental rotation (MR). We found that higher WM scores significantly correlated with better gait performance under dual-task conditions, including faster gait velocity (r = 0.6524, p = 0.0025), longer stride length (r = 0.6758, p = 0.0015), higher toe clearance (r = 0.5234, p = 0.0215), and greater hip range of motion (ROM) (r = 0.6803, p = 0.0014). Similarly, better MR ability was associated with longer stride length (r = 0.5178, p = 0.0231) and greater hip ROM (r = 0.4886, p = 0.0338) during dual-task walking. Furthermore, superior WM and MR performance were linked to faster TUG completion times, particularly during the second walking phase of the TUG (WM: r = −0.682, p = 0.0013; MR: r = −0.6755, p = 0.002). These results indicate that WM and MR may be linked to gait performance in PD, especially during cognitively challenging walking tasks. These findings may have clinical, rehabilitative, and neuroscientific utility for those involved in the study and management of PD.

## Introduction

Parkinson disease (PD) is the second most common neurodegenerative disease worldwide [1] with projected increased prevalence through the next two decades [2]. Current evidence suggests PD results from a confluence of genetic and environmental factors that cause abnormal α-synuclein protein accumulation, neuroinflammation, and cellular dysfunction [3,4]. The evidence of neuropathology, both centrally and peripherally, has generated diverging theories regarding the localization of disease onset [5,6] Early loss of dopaminergic neurons in the nigrostriatal pathway underlies the hallmark motor symptom of bradykinesia [7,8]. Neurodegenerative processes begin decades before diagnosis [9] with subtle symptoms developing in the prodromal stage and overt signs presenting in the manifestation stage. Parkinson disease is primarily characterized by motor dysfunction, but non-motor symptoms are equally present and cause considerable morbidity and disability. Autonomic dysregulation, sleep disorders, depression, and cognitive impairment are significant contributors to disease burden [4].

Gait dysfunction is a cardinal biomarker of PD. Bradykinesia, reduced joint range of motion (ROM), and postural instability are characteristic signs of PD. In comparison to healthy controls, individuals with PD display multiple spatiotemporal and kinematic differences such as slower gait velocity, shorter step and stride lengths, alterations of cadence, and asymmetrical arm movement [10,11]. Gait changes may become more pronounced in later stages of PD (i.e., festinating or freezing of gait) reflecting degeneration in central locomotor networks [12–15]. Healthy gait relies on motor automaticity, requiring minimal attentional output from frontal cortical areas. Motor automaticity is impaired in PD (review by Wu et al. [16]) as feedforward gait programs become deficient secondary to pathological nigrostriatal-thalamo-cortico loops. Individuals with PD compensate for these neural deficiencies by increasing activation of attentional fronto-striatal networks during locomotion [17]. However, the additional cognitive load, particularly during dual-task (DT) gait, can fatigue fronto-striatal and forebrain cholinergic regions [17,18]. These neurodegenerative processes begin early in the silent stage of PD and are progressive. Although non-specific, gait is a practical clinical biomarker for detection and monitoring of PD [19–21].

The Timed Up and Go (TUG) task is a widely used tool for musculoskeletal and neurological examination, including for individuals with PD [22,23]. The components of vertical transitions (*i.e.*, sit-to stand, stand-to-sit), walking, turning, and spatial planning involve both motor and cognitive efforts. Phase analysis of the TUG task has revealed locomotor deficits that are not evident in routine walking [24]. For example, freezing-of-gait may be expressed during the turning sub-phase of TUG but not in other sub-phases [25].

The level of cognitive impairment (CI) is heterogenous in individuals with PD. In their frequently cited study, Buter et al. [26] reported 80% of patients with PD will develop dementia over the course of disease progression. However, recent authors [27] have argued the incidence of dementia in PD is significantly lower (~50% over 15 years). Nevertheless, CI is recognized as a prevalent non-motor concomitant that may occur in early stages of PD [28] and progress to dementia in susceptible individuals. Cognitive impairments of attention, language, and memory are associated with a reduction in daily activities independent of motor disability [29].

Working memory (WM) is a cognitive task involving multiple neural substrates and circuits. WM localizes most notably to the dorsolateral prefrontal cortex, frontal eye fields, and inferior parietal lobe with direct connections to the caudate [30]. Introductory work by Owen et al. [31] and more recently Hattori et al. [30] have explored the cognitive demand of WM in PD, revealing that patients without overt cognitive impairment require increased neural activation to accomplish simple WM tasks. Furthermore, patients with more advanced cognitive deficits often cannot meet the neural demand as task difficulty increases.

Response inhibition (RI) is a cognitive-motor task often measured with Stroop tasks or go-no-go paradigms. Neural correlates of RI are predominantly frontal and include the prefrontal cortex, anterior cingulate cortex, and the subthalamic nucleus of basal ganglia [32,33]. These structures are inherent to the fronto-striatal-thalamo-cortical circuits susceptible to neurodegeneration and denervation present in PD. Deficiencies of the noradrenergic locus coeruleus is associated with CI and correlates with poor RI performance by individuals with PD [34,35]. Response inhibition has been used as an evaluation tool in the PD population as a surrogate biomarker of psychological and/or motor inhibition (see review by MacDonald et al. [36]).

Mental rotation (MR) is a visuospatial cognitive task localizing to posterior cortical regions with parallel anterior feedforward contribution. Superior and inferior parietal lobes, visual cortices, ventral and dorsal visual pathways are the principal neural substrates [37,38]. Importantly, there is also pre-motor and cerebellar involvement as MR requires motor planning [37]. Visuospatial dysfunction is evident in PD [39] and MR is a unique challenge due to its pre-motor planning and imagery requirements [40]. Furthermore, MR deficits in PD may indicate neurodegeneration beyond dopaminergic networks [41].

Gait and cognition share neural substrates particularly in nigro-striatal-thalamo-cortical circuits. In PD, motor-cognitive skills create demands that may overwhelm susceptible neural circuitry resulting in motor faults, cognitive dysfunction, or both. Although gait disturbances during cognitive DT efforts are well reported in the PD population, very few studies have reported the relationship between specific cognitive skills and gait performance in PD. Penko, et al. [42,43] found that individuals with PD walked slower with decreased stride length and joint ROM during cognitive DT, particularly during a WM challenge. Harrie et al. [44] reported TUG phase times under DT conditions correlated strongly with visuospatial and processing skills, more so than executive functions. Amboni, et al. [45] performed a correlation analysis of neuropsychological tests and gait, revealing strong correlation of global cognition with multiple gait parameters (i.e., cadence, velocity, support time). However, visuospatial orientation was the only cognitive domain significantly correlated with a specific gait parameter (velocity).

In this study, we explore correlations between distinct cognitive domains and detailed gait kinematics in individuals with PD. Using marker-less motion capture technology and validated neuropsychological tasks, we aim to reveal the relationships of WM, RI, and MR with spatiotemporal gait parameters during ST walking, DT walking, and TUG phase times. Clarifying these relationships can improve our understanding of specific motor-cognitive interactions in PD and guide more precise clinical and rehabilitative management strategies. Furthermore, these findings may be useful for neuroscience researchers investigating PD circuit pathophysiology.

## Materials and methods

All study procedures were approved by the University’s Institutional Review Board and conducted in accordance with the Declaration of Helsinki. All participants were required to have a diagnosis of PD and be able to ambulate without assistance. Additionally, all participants met the following criteria: (i) Free of any history of ancillary orthopedic conditions that precluded walking (*e.g.*, acute injury, recent surgery) (ii) Free of any other neurological disorders with concomitant CI (*e.g.*, stroke), (iii) Maintenance of normal medication schedule. There were no exclusion criteria related to sex or baseline cognitive status. Twenty-one participants (15 females, 6 males; 65.7 ± 5.7 yo), were enrolled from a referral sample of local PD support group members and a convenience sample from a private medical practice. All participants met the above criteria and volunteered for this investigation.

### Initial screening and assessment

After participants volunteered for study, a focused health history was taken to obtain demographic information and confirm candidacy. Motor function was evaluated using part three (motor) of the Movement Disorder Society-Unified Parkinson’s Disease Rating Scale (MDS-UPDRS) [46]; Patient Health Questionnaire (PHQ-9) [47] and the Montreal Cognitive Assessment (MoCA) were completed, respectively. Participants maintained their normally prescribed medication schedule. As standard, levodopa equivalent daily dose (LEDD) was calculated for each participant [48]. For all cognitive assessments, individuals used corrective lenses as needed. No participants reported color visual deficits. All assessments were conducted by one of the study’s primary investigators (JJL).

### Computer neuropsychological assessment (CNA)

Participants completed three CNA domain tasks from Creyos web-based software (Toronto, ON, CA) in a controlled environment. Participants were seated in a quiet setting, utilized a tablet computer (Apple iPad, Cupertino, CA) and were provided verbal instructions. Data was collected via an online portal (www.creyos.com). Creyos software offers easy to use gamified modes that are engaging to the user. Along with verbal instructions, Creyos software provided individualized instructions prior to each assessment with a series of practice trials. No instructions or assistance were provided during the assessments. Domain tasks of working memory (‘Token Search’), mental rotation/visuospatial processing (‘Rotations’), and attention/response inhibition Stroop task (‘Double Trouble’) were assessed. The WM task involved remembering the location of a hidden token in a group of boxes. Participants were challenged to find the token by tapping individual boxes and remembering which boxes were already selected. Choosing the same box twice in a round resulted in a penalty. Each successful round increased the difficulty in the following round. Three total penalties ended the game. To assess visuospatial processing, a timed task (90 seconds) of MR was used. Participants were charged with mentally rotating a grid of shapes to discern if it was identical to a corresponding grid. Participants selected if the grids were a “match” or “no match”. The game progressed in difficulty with each successful round. Finally, attention/response inhibition was assessed via a timed Stroop task (90 seconds). The Stroop task included word-color pairs of varied congruencies (congruent-congruent, incongruent-congruent, incongruent-incongruent). Participants were charged with selecting the appropriate word-color match. These cognitive tasks are commonly used with various demographics and have previously assessed cognition in PD patients [31,49,50]. Creyos software has norm-based age/sex scoring, has adequate concurrent validity when compared to in-person neuropsychological assessment, marginal-to-low test-retest reliability depending on specific test item, and good usability among older adults with cognitive decline [51,52].

### Experimental protocol

Participants performed two TUG assessments and four 15-meter overground walking trials with a single task (ST) and dual task (DT) condition. Participants wore their preferred closed toe walking footwear and self-selected attire. For the TUG assessment, participants were instructed to stand from a seated position, walk three meters before turning 180^o^ around a mark taped to the floor, walk back to chair, turn, and sit down. The TUG assessment has been previously reported as having high test-retest reliability in individuals with PD [23]. Participants then performed two ST walking trials where they were instructed to walk at a self-selected pace for 15 meters. Sport cones marked the beginning and end of the 15-meter walkway. To conclude the protocol, participants performed two DT walking trials, when they were given a word to spell backward while they walked at their self-selected pace. Backward spelling was used in lieu of the common DT of serial 7 subtraction to avoid potential learning effects from previous exposure as the subtraction task is often used in the clinical setting. Hassan, et al. [53] have reported backward spelling as an effective dual-task cognitive load for older adults. One five-letter word for each trial (1 -“Radio”, 2 -“Baker”) was provided to participants within the first two steps that began the trial. No other verbal instructions were provided, a practice trial was afforded for each ST test condition (TUG, overground walking), and adequate recovery (~60 seconds) was granted between all trials.

### Biomechanical assessment

**Fig 1:**
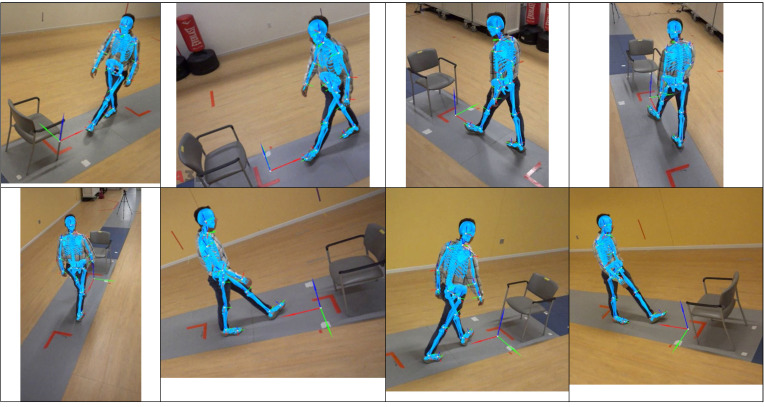
An eight-camera motion capture system (120 Hz) captured synchronized video of all trials to allow three-dimensional tracking a 19-segment kinematic model within Theia3D markerless motion capture software (Theia Markerless Inc., Kingston, ON, CA).

An eight-camera video system (Sony RXO II; Sony Corporation; Minato, Japan) recorded trials at 120 frames per second. Synchronized videos were calibrated to estimate a 19-segment kinematic model with a commercial marker-less (ML) motion capture software package (v2023.1.03161 patch 11; Theia Markerless Inc., Kingston, ON, CA). The ML motion capture system utilizes deep learning neural networks to estimate three-dimensional pose estimates (Fig 1) with a more detailed explanation of pose determination provided at Kanko et al. [54]. Previous investigations have reported good-to-excellent agreement for the determination of spatiotemporal metrics of walking gait (e.g., velocity, stride length) using this methodology when compared to a pressure-sensitive mat [54,55]. Additionally, sagittal plane joint kinematics were reported to have excellent agreement (RMS < 4^o^ degrees) with marker-based motion capture [56].

Kinematic data (4x4 segment rotation matrices) and locations of virtual bilateral heel markers were exported and subsequently processed with Visual3D Professional (C-Motion Inc.; Germantown, MD, USA; v2023.12.1). Data was filtered with a GCVSPL filter with 12 Hz cutoff frequency GCVSPL filter [57]. A Visual3D segmental model with physical properties (segmental mass, center of mass, center of mass location) derived from previous work [58–60] was used to estimate and track full body center of mass (COM) location.

### Data processing

For both TUG trials, the COM’s x-coordinate (direction of travel) and z-coordinate (superior-inferior) respective positions (Table 1) were utilized to determine the relevant phase times for each trial. Component TUG times were measured for the following phases: (1) sit-to-stand, (2) gait initiation, (3) walk 1, (4) turn, (5) walk 2, (6) slow down & turn, and (7) sit down. These component tasks are similar to a past report that partitioned the TUG into requisite phases [61]. Similarly, COM location was also tracked during a gait initiation task within a group of individuals with PD, with the same ML mocap system used in the current study and deemed to efficiently detect motor performance [62].

**Table 1:** Full body center of mass (COM) x- and z-coordinates were utilized to automatically determine timing of event markers within the timed up and go (TUG) assessment.

Event Marker	Description of Automatic Detection
Initiation of Stand Phase	COM z-position > starting z-position
End of Stand Phase	First maximum peak of COM z-position following stand initiation
End of Gait Initiation Phase	COM x-position > 1 m beyond starting COM position
Begin Turn Phase	COM x-position > 3 m of starting COM position
End Turn	COM x-position < 3 m of starting COM position
End of Walk 2	COM x-position < 1 m of starting COM position
Begin Sit	COM z-position < 95% COM z-position from standing position
End Sit	COM x-position ≤ starting COM x-position

For all overground walking trials, a 3.3-meter zone within the center of the calibrated three-dimensional volume was used to demarcate the beginning and end of trial respectively. Location of COM was used to determine these respective endpoints with walking velocity computed for this interval. All participants had > 5m walking prior to this collection zone to avoid gait initiation phase and allowing for at least two strides within capture period. Stance phase event markers (foot strike, foot off) were determined via a coordinate-based algorithm of the heel and toe marker relative to the body’s COM. This is a similar method to a previous gait investigation using the same ML mocap system [55]. Based on these gait events, stride length (SL) and double support (DS) time were computed and averaged for each trial. Additionally, minimum toe clearance (TC) during swing phase was determined bilaterally for each trial. Hip and shoulder flexion-extension joint angles were computed in Visual3D utilizing an XYZ Cardan rotation sequence consistent with a standardized joint coordinate system [63]. Bilateral hip and shoulder ROM during the gait cycle were determined bilaterally for each trial. All TUG phase times, and biomechanical walking gait metrics (SL, DS, TC, Hip ROM, and shoulder ROM) were exported to a custom Python (ver. 3.12.4) script for further data analysis and statistical processing. As arm swing asymmetry during gait has been reported to be increased in PD [19], the symmetry angle [64] was computed between bilateral shoulder ROM metrics. For statistical processing, only the toe clearance and hip ROM from the impaired side was used for analysis. For any participant with bilateral impairments (n = 1) or no impairments (n = 1), the average across sides was used for analysis.

### Statistical analysis

All dependent variables were first assessed for normality with the Shapiro-Wilk test to determine the appropriate correlation test. Pairwise correlations were conducted between (i) all cognitive task metrics and TUG metrics, and (ii) all cognitive task metrics and walking metrics using either Pearson or Spearman correlation depending on the results of normality test. Both the normality and correlation tests were computed utilizing the SciPy library [65] within Python. Finally, an ordinary least squares (OLS) regression and a multivariate analysis of variance (MANOVA) were performed to evaluate the overall effect of cognitive task scores on the TUG and walking metrics, respectively. These analyses were conducted using the statsmodels library [66] in Python.

## Results

Data from nineteen participants was used for subsequent analysis with two participants excluded for either not completing all sessions of protocol or methodological issues related to video file storage. The mean age was 65.7 ± 5.7 years. Participants had mild motor symptoms on average with a mean UPDRS Part III score 11.4 ± 8.9, indicating early-stage PD. Education level was high with a median of 16 years completed and 43% of participants achieving graduate degrees. The median MoCA score was 27 with one participant meeting the criteria suggestive of mild cognitive impairment. All participants maintained their PD medication schedule (two participants were non-medicated). The mean LEDD 446 ± 279 mg (range 0–1080 mg). Complete descriptive statistics of participant demographics (Table 2) and dependent variables were computed with accompanying normality checks (Table 3). All variables were normally distributed (*p* > 0.05) except for RI (*p* = 0.031), TUG sit down phase time (*p* = 0.003), shoulder asymmetry ST (*p* = 0.005), double support DT (*p* = 0.026) and shoulder asymmetry DT (*p* = 0.004). Additionally, the variation inflation factor (VIF) for all cognitive skill task scores were < 2.7, indicating that multicollinearity of variables would not influence regression findings.

**Table 2:** Demographic variables of the nineteen participants with Parkinson Disease who completed all aspects of protocol with data being used for statistical analysis.

Demographic Variable	Mean (M)	Standard Deviation (SD)	Minimum (Min)	Maximum (Max)	Median	Frequency (N)	Percentage (%)
Age	66.2	5.8	57	81	65		
Sex							
- Male						6	31.6%
- Female						13	68.4%
Education (yrs)	12	6	6	18	16		
- Grade School						1	5.0%
- High School						1	5.0%
− 2 yr College						0	0%
− 4 yr College						8	38%
- Graduate School						9	43%
Years Since Diagnosis	5.4	4.7	1	18	4		
Hoehn & Yahr Scale	2.3	0.3	1	3	2.3		
UPDRS (Part 3)	11.4	8.9	2	40	7		
LEDD(mg)	446.4	279.4	0	1080	375		
MoCA	26	3.3	19	30	27		
PHQ-9	4.3	3.3	0	14	3		

Note: UPDRS, Unified Parkinsons Disease Rating Scale; LEDD, Levodopa Equivalent Daily Dose; MoCA, Montreal Cognitive Assessment; PHQ-9, Patient Health Questionnaire.

**Table 3:** Descriptive statistics (Mean, Standard Deviation (Std)) and normality test scores for dependent variables.

Metric	Mean	Std	Shapiro-Wilk Statistic	p-value	Normality
**Cognitive Test Scores**					
Response Inhibition	13.0	13.9	0.889	0.031	FALSE
Working Memory	4.7	1.2	0.963	0.642	TRUE
Mental Rotation	54.1	31.3	0.943	0.302	TRUE
**TUG Phase Times (s)**					
Sit-to-stand	1.13	0.30	0.905	0.061	TRUE
Gait initiation	0.59	0.19	0.930	0.174	TRUE
Walk 1	1.73	0.27	0.971	0.802	TRUE
Turn	2.98	0.68	0.962	0.617	TRUE
Walk 2	1.89	0.38	0.951	0.407	TRUE
Slow down & turn	1.32	0.44	0.954	0.467	TRUE
Sit down	1.33	0.56	0.831	0.003	FALSE
**Overground Walking**					
**Single Task (ST)**					
Velocity (m/s)	1.32	0.21	0.960	0.580	TRUE
Stride Length (m)	1.36	0.19	0.968	0.738	TRUE
Double Support (s)	0.34	0.03	0.939	0.249	TRUE
Toe Clearance (m)	0.034	0.011	0.960	0.563	TRUE
Hip ROM (deg)	50.38	6.11	0.935	0.218	TRUE
Shoulder Asymmetry (%)	9.03	8.30	0.843	0.005	FALSE
**Dual Task (DT)**					
Velocity (m/s)	1.12	0.28	0.951	0.416	TRUE
Stride Length (m)	1.22	0.22	0.958	0.526	TRUE
Double Support (s)	0.40	0.05	0.885	0.026	FALSE
Toe Clearance (m)	0.030	0.012	0.943	0.301	TRUE
Hip ROM (deg)	46.42	8.37	0.926	0.148	TRUE
Shoulder Asymmetry (%)	12.85	13.25	0.838	0.004	FALSE

Note: TUG = timed up and go; ROM = range of motion.

### Timed up & go test (TUG)

**Fig 2:**
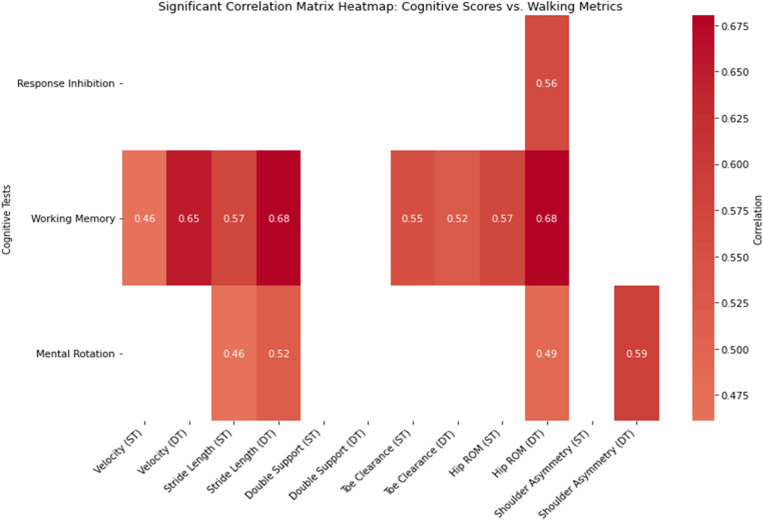
Correlation heat map depicting strength of significant correlations between timed up and go phase times with cognitive test scores.

The sit-to-stand phase time was negatively correlated with RI (r = −0.4704, p = 0.042), WM (r = −0.613, p = 0.005) and MR (r = −0.5401, p = 0.017) cognitive skills respectively (Table 4, Fig 2). The Walk 1 phase time was negatively correlated with

**Table 4:** Pairwise correlations were individually run between predictor cognitive variables (Response Inhibition, Working Memory and Mental Rotations) with each respective timed up and go (TUG) phase time metric. Asterisks (*) indicate significant correlations (p < 0.05).

Cognitive Test	TUG Phase	Correlation	p-value	95% CI (Lower)	95% CI (Upper)
Response Inhibition	Sit-to-Stand	−0.4704*	0.0421	−0.7781	0.0193
	Gait Initiation	0.0705	0.7744	−0.4296	0.5374
	Walk 1	−0.4861*	0.0348	−0.7860	−0.0010
	Turn	−0.4366	0.0616	−0.7608	0.0618
	Walk 2	−0.5114*	0.0252	−0.7985	−0.0346
	Slow Down & Turn	−0.2148	0.3773	−0.6340	0.3021
	Sit Down	−0.4102	0.0811	−0.7469	0.0939
Working Memory	Sit-to-Stand	−0.613*	0.0053	−0.8465	−0.1817
	Gait Initiation	0.1025	0.6764	−0.4029	0.5600
	Walk 1	−0.5186*	0.0229	−0.8021	−0.0445
	Turn	−0.5592*	0.0128	−0.8216	−0.1013
	Walk 2	−0.682*	0.0013	−0.8771	−0.2940
	Slow Down & Turn	−0.4619*	0.0465	−0.7738	0.0302
	Sit Down	−0.4191	0.0741	−0.7516	0.0831
Mental Rotations	Sit-to-Stand	−0.5401*	0.017	−0.8125	−0.0742
	Gait Initiation	−0.1389*	0.5706	−0.5849	0.3715
	Walk 1	−0.6458*	0.0028	−0.8612	−0.2338
	Turn	−0.6377*	0.0033	−0.8576	−0.2206
	Walk 2	−0.6755*	0.0015	−0.8742	−0.2829
	Slow Down & Turn	−0.4989*	0.0297	−0.7924	−0.0179
	Sit Down	−0.2917	0.2257	−0.6807	0.2256

RI (r = −0.4861, p = 0.035), WM (r = −0.5186, p = 0.023), and MR (r = −0.6458, p = 0.003) cognitive skills respectively. The turning phase time was negatively correlated with RI (r = −0.4366, p = 0.062), WM (r = −0.5592, p = 0.013), and MR (r = −0.6377, p = 0.003) cognitive skills respectively. The Walk 2 phase time was negatively correlated with RI (r = −0.5114, p = 0.025), WM (r = −0.6820, p = 0.001), and MR (r = −0.6755, p = 0.002) cognitive skills respectively. The slow down & turn phase time was negatively correlated with WM (r = −0.4619, p = 0.047) and MR (r = −0.4989, p = 0.030), but showed no significant correlation with RI (r = −0.2148, p = 0.377). Both the gait initiation and sit down phases had no significant correlation with any of the cognitive skills.

The OLS regression models explained significant portions of variance for each TUG phase: Sit to Stand (44.4%), Gait Initiation (7.1%), Walk 1 (45.8%), Turn Around (48.8%), Walk 2 (61.9%), Slow Down & Turn (41.1%), and Sit Down (19.8%) (Table 5). All TUG phases exhibited significant intercepts (p < 0.05) in the OLS regression models, indicating substantial baseline phase times independent of cognitive skill metrics. Mental rotation performance was significantly associated with Walk 1 phase time (B = −0.005, p = 0.048), Turn phase time (B = −0.012, p = 0.039), and Walk 2 phase time (B = −0.006, p = 0.027) respectively. Additionally, WM was significantly associated with Walk 2 phase time (B = −0.184, p = 0.029).

**Table 5:** Ordinary least squares multivariable regression results between timed up and go phase times and cognitive test scores.

TUG Phase	Predictor	B	SE	t	p	95% CI (Lower)	95% CI (Upper)	R²	Adj. R²
Sit-to-Stand	Intercept	1.886	0.292	6.458	<0.001*	1.264	2.508	0.444	0.332
	Response Inhibition	0.003	0.007	0.415	0.684	−0.012	0.017		
	Working Memory	−0.132	0.073	−1.805	0.091	−0.288	0.024		
	Mental Rotations	−0.003	0.002	−1.348	0.198	−0.008	0.002		
Gait Initiation	Intercept	0.54	0.243	2.223	0.042*	0.022	1.057	0.071	−0.115
	Response Inhibition	0.002	0.006	0.336	0.741	−0.01	0.014		
	Working Memory	0.028	0.061	0.453	0.657	−0.102	0.157		
	Mental Rotations	−0.002	0.002	−0.987	0.339	−0.006	0.002		
Walk 1	Intercept	2.236	0.261	8.583	<0.001*	1.681	2.792	0.458	0.349
	Response Inhibition	0.001	0.006	0.082	0.936	−0.012	0.013		
	Working Memory	−0.057	0.065	−0.876	0.395	−0.196	0.082		
	Mental Rotations	−0.005	0.002	−2.148	0.048*	−0.009	0		
Turn	Intercept	4.597	0.632	7.271	<0.001*	3.249	5.944	0.488	0.386
	Response Inhibition	0.01	0.015	0.707	0.49	−0.021	0.041		
	Working Memory	−0.241	0.158	−1.517	0.15	−0.578	0.097		
	Mental Rotations	−0.012	0.005	−2.265	0.039*	−0.023	−0.001		
Walk 2	Intercept	2.999	0.305	9.838	<0.001*	2.35	3.65	0.619	0.542
	Response Inhibition	0.006	0.007	0.906	0.379	−0.009	0.021		
	Working Memory	−0.184	0.076	−2.408	0.029*	−0.347	−0.021		
	Mental Rotations	−0.006	0.002	−2.458	0.027*	−0.011	−0.001		
Slow Down & Turn	Intercept	2.488	0.437	5.695	<0.001*	1.557	3.418	0.411	0.293
	Response Inhibition	0.017	0.01	1.671	0.115	−0.005	0.038		
	Working Memory	−0.213	0.109	−1.943	0.071	−0.446	0.021		
	Mental Rotations	−0.007	0.004	−2.011	0.063	−0.015	0		
Sit Down	Intercept	2.009	0.653	3.076	0.008*	0.617	3.401	0.198	0.037
	Response Inhibition	−0.008	0.015	−0.546	0.593	−0.04	0.024		
	Working Memory	−0.116	0.164	−0.708	0.49	−0.465	0.233		
	Mental Rotations	−0.001	0.005	−0.104	0.919	−0.012	0.011		

The MANOVA results indicated that cognitive scores were collectively associated with the TUG performance (Table 6). The overall model was significant (Wilks’ lambda = 0.0725, F(7, 9) = 16.4564, p = 0.0002). Specifically, WM was significantly associated with the TUG phase times (Wilks’ lambda = 0.2485, F(7, 9) = 3.8886, p = 0.0313), whereas RI and MR did not reach significance (p > 0.05).

**Table 6:** MANOVA results for TUG data.

Predictor	Test	Value	F Value	Num DF	Den DF	p-Value
**Intercept**	Wilks’ lambda	0.0725	16.4564	7	9	0.0002
	Pillai’s trace	0.9275	16.4564	7	9	0.0002
	Hotelling-Lawley trace	12.7994	16.4564	7	9	0.0002
	Roy’s greatest root	12.7994	16.4564	7	9	0.0002
**Response Inhibition**	Wilks’ lambda	0.3241	2.6816	7	9	0.0849
	Pillai’s trace	0.6759	2.6816	7	9	0.0849
	Hotelling-Lawley trace	2.0857	2.6816	7	9	0.0849
	Roy’s greatest root	2.0857	2.6816	7	9	0.0849
**Working Memory**	Wilks’ lambda	0.2485	3.8886	7	9	0.0313
	Pillai’s trace	0.7515	3.8886	7	9	0.0313
	Hotelling-Lawley trace	3.0244	3.8886	7	9	0.0313
	Roy’s greatest root	3.0244	3.8886	7	9	0.0313
**Mental Rotations**	Wilks’ lambda	0.4366	1.659	7	9	0.2351
	Pillai’s trace	0.5634	1.659	7	9	0.2351
	Hotelling-Lawley trace	1.2903	1.659	7	9	0.2351
	Roy’s greatest root	1.2903	1.659	7	9	0.2351

### Single task (ST) walking

**Fig 3:**
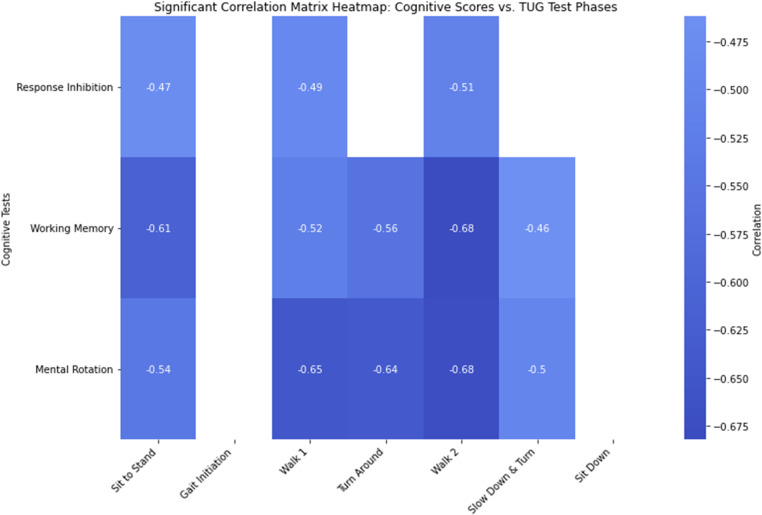
Correlation heat map depicting strength of significant correlations between walking metrics from both single task (ST) and dual task (DT) with cognitive test scores.

During the ST walking condition, WM demonstrated a significant positive correlation with velocity (r = 0.4619, p = 0.0465), stride length (r = 0.5707, p = 0.0107), toe clearance (r = 0.5517, p = 0.0143), and hip ROM (r = 0.5715, p = 0.0106). The only other significant correlation during the ST walking condition was between MR and stride length (r = 0.4610, p = 0.0470) (Table 7, Fig 3).

**Table 7:** Pairwise correlations were individually run between predictor cognitive variables (Response Inhibition, Working Memory and Mental Rotations) with each respective single task (ST) walking metric. Asterisks (*) indicate significant correlations (p < 0.05).

Predictor	Dependent Variable	Correlation	p-value	95% CI (Lower)	95% CI (Upper)
Response Inhibition	Velocity (ST)	0.2267	0.351	−0.2907	0.6415
	Stride Length (ST)	0.2969	0.217	−0.2202	0.6837
	Double Support (ST)	0.0924	0.707	−0.4114	0.5530
	Toe Clearance (ST)	0.2705	0.263	−0.2473	0.6682
	Hip ROM (ST)	0.2744	0.256	−0.1853	0.7026
	Shoulder Asymmetry % (ST)	0.2132	0.381	−0.3036	0.6331
Working Memory	Velocity (ST)	0.4619	0.0465*	−0.0303	0.7738
	Stride Length (ST)	0.5707	0.0107*	0.1180	0.8270
	Double Support (ST)	0.1697	0.487	−0.3440	0.6052
	Toe Clearance (ST)	0.5517	0.0143*	0.1179	0.8269
	Hip ROM (ST)	0.5715	0.0106*	0.0196	0.7930
	Shoulder Asymmetry % (ST)	0.0156	0.950	−0.4734	0.4972
Mental Rotations	Velocity (ST)	0.2694	0.265	−0.2484	0.6675
	Stride Length (ST)	0.4610	0.0470*	−0.0313	0.7733
	Double Support (ST)	0.0717	0.771	−0.4286	0.5383
	Toe Clearance (ST)	0.2887	0.231	−0.2287	0.6790
	Hip ROM (ST)	0.3605	0.130	−0.2397	0.6726
	Shoulder Asymmetry % (ST)	0.3991	0.091	−0.1070	0.7409

The OLS regression models explained portions of variance for each walking metric under single-task conditions: Velocity (25.4%), Stride Length (43.9%), Double Support (3.2%), Toe Clearance (34.2%), Hip ROM (34.2%), and Shoulder Asymmetry (23.6%) (Table 8). Most walking metrics exhibited significant intercepts (p < 0.05) in the OLS regression models, indicating substantial baseline phase times independent of cognitive skill metrics. However, the intercepts for toe clearance (p = 0.684) and shoulder asymmetry (p = 0.166) were not significant. Working memory performance was significantly associated with stride length (B = 0.1119, p = 0.027) and toe clearance (B = 0.0064, p = 0.028). Additionally, WM was associated with Hip ROM (B = 3.0743, p = 0.063).

**Table 8:** Ordinary least squares regression analysis examining the relationship between single-task (ST) walking metrics and cognitive test scores.

Walking Metric	Predictor	B	SE	t	p	95% CI (Lower)	95% CI (Upper)	R²	Adj. R²
Velocity (ST)	Intercept	0.8196	0.235	3.484	0.003*	0.318	1.321	0.254	0.105
	Response Inhibition	−0.0049	0.005	−0.904	0.381	−0.016	0.007		
	Working Memory	0.1109	0.059	1.881	0.08	−0.015	0.237		
	Mental Rotations	0.0008	0.002	0.435	0.67	−0.003	0.005		
Stride Length (ST)	Intercept	0.8003	0.182	4.405	0.001*	0.413	1.188	0.439	0.326
	Response Inhibition	−0.0061	0.004	−1.465	0.164	−0.015	0.003		
	Working Memory	0.1119	0.046	2.456	0.027*	0.015	0.209		
	Mental Rotations	0.0021	0.001	1.416	0.177	−0.001	0.005		
Double Support (ST)	Intercept	0.313	0.044	7.169	<0.001*	0.22	0.406	0.032	−0.162
	Response Inhibition	−0.0002	0.001	−0.181	0.858	−0.002	0.002		
	Working Memory	0.0065	0.011	0.592	0.563	−0.017	0.03		
	Mental Rotations	0	0	−0.02	0.985	−0.001	0.001		
Toe Clearance (ST)	Intercept	0.0043	0.01	0.415	0.684	−0.018	0.027	0.342	0.211
	Response Inhibition	−0.0002	0	−0.823	0.423	−0.001	0		
	Working Memory	0.0064	0.003	2.429	0.028*	0.001	0.012		
	Mental Rotations	0	0	−0.095	0.925	0	0		
Hip ROM (ST)	Intercept	36.144	6.106	5.919	<0.001*	23.129	49.159	0.342	0.211
	Response Inhibition	−0.0744	0.141	−0.53	0.604	−0.374	0.225		
	Working Memory	3.0743	1.531	2.009	0.063	−0.188	6.337		
	Mental Rotations	0.0221	0.049	0.448	0.661	−0.083	0.128		
Shoulder Asymmetry % (ST)	Intercept	13.8085	9.481	1.456	0.166	−6.4	34.017	0.236	0.083
	Response Inhibition	0.1345	0.218	0.616	0.547	−0.331	0.6		
	Working Memory	−2.8752	2.377	−1.21	0.245	−7.941	2.19		
	Mental Rotations	0.1287	0.077	1.677	0.114	−0.035	0.292		

The MANOVA results indicated that cognitive scores were collectively associated with the walking performance in the single-task condition (Table 9). The overall model was significant (Wilks’ lambda = 0.0339, F(6, 10) = 47.5500, p < 0.0001). Specifically, WM was significantly associated with the walking metrics (Wilks’ lambda = 0.3420, F(6, 10) = 3.2070, p = 0.0504), whereas RI and MR did not reach significance (p > 0.05).

**Table 9:** Multivariate analysis of variance (MANOVA) results assessing the impact of cognitive test scores on walking metrics under single-task (ST) conditions.

Predictor	Test	Value	F Value	Num DF	Den DF	p-Value
**Intercept**	Wilks’ lambda	0.0339	6	10	47.55	< 0.001*
	Pillai’s trace	0.9661	6	10	47.55	< 0.001*
	Hotelling-Lawley trace	28.53	6	10	47.55	< 0.001*
	Roy’s greatest root	28.53	6	10	47.55	< 0.001*
**Response Inhibition**	Wilks’ lambda	0.7451	6	10	0.5701	0.7462
	Pillai’s trace	0.2549	6	10	0.5701	0.7462
	Hotelling-Lawley trace	0.3421	6	10	0.5701	0.7462
	Roy’s greatest root	0.3421	6	10	0.5701	0.7462
**Working Memory**	Wilks’ lambda	0.342	6	10	3.207	0.0504
	Pillai’s trace	0.658	6	10	3.207	0.0504
	Hotelling-Lawley trace	1.9242	6	10	3.207	0.0504
	Roy’s greatest root	1.9242	6	10	3.207	0.0504
**Mental Rotations**	Wilks’ lambda	0.6027	6	10	1.0985	0.4259
	Pillai’s trace	0.3973	6	10	1.0985	0.4259
	Hotelling-Lawley trace	0.6591	6	10	1.0985	0.4259
	Roy’s greatest root	0.6591	6	10	1.0985	0.4259

### Dual task (DT) walking condition

During the DT walking condition, RI demonstrated a significant positive correlation with hip ROM (r = 0.5632, p = 0.0120). Working memory demonstrated a significant positive correlation with velocity (r = 0.6524, p = 0.0025), stride length (r = 0.6758, p = 0.0015), toe clearance (r = 0.5234, p = 0.0215), and hip ROM (r = 0.6803, p = 0.0014). Additionally, MR showed significant positive correlations with stride length (r = 0.5178, p = 0.0231), hip ROM (r = 0.4886, p = 0.0338) and shoulder asymmetry (r = 0.5894, p = 0.0079) (Table 10, Fig 3

**Table 10:** Pairwise correlations were individually run between predictor cognitive variables (Response Inhibition, Working Memory and Mental Rotations) with each respective dual task (DT) walking metric. Asterisks (*) indicate significant correlations (p < 0.05).

**Predictor**	**Dependent Variable**	**Correlation**	**p-value**	**95% CI (Lower)**	**95% CI (Upper)**
Response Inhibition	Velocity (DT)	0.4179	0.075	−0.0846	0.7509
	Stride Length (DT)	0.3555	0.1353	−0.1569	0.7171
	Double Support (DT)	−0.1477	0.5462	−0.6939	0.2016
	Toe Clearance (DT)	0.3413	0.153	−0.1733	0.7089
	Hip ROM (DT)	0.5632	0.0120*	0.0992	0.8209
	Shoulder Asymmetry (DT)	0.1337	0.5852	−0.3760	0.5814
Working Memory	Velocity (DT)	0.6524	0.0025*	0.2445	0.8642
	Stride Length (DT)	0.6758	0.0015*	0.2833	0.8743
	Double Support (DT)	−0.3145	0.1897	−0.6939	0.2016
	Toe Clearance (DT)	0.5234	0.0215*	0.0480	0.8033
	Hip ROM (DT)	0.6803	0.0014*	−0.6939	0.2016
	Shoulder Asymmetry (DT)	0.2411	0.3201	−0.2766	0.6504
Mental Rotations	Velocity (DT)	0.3583	0.132	−0.1538	0.7187
	Stride Length (DT)	0.5178	0.0231*	0.0434	0.8017
	Double Support (DT)	−0.1418	0.5627	−0.5867	0.3690
	Toe Clearance (DT)	0.3514	0.140	−0.1911	0.6996
	Hip ROM (DT)	0.4886	0.0338*	−0.0097	0.7819
	Shoulder Asymmetry (DT)	0.5894	0.0079*	0.1457	0.8357

The OLS regression models explained significant portions of variance for each walking metric under dual-task conditions: velocity (43.9%), stride length (58.9%), double support (11.6%), toe clearance (28.7%), hip ROM (48.4%), and shoulder asymmetry (44.4%) (Table 11). Most walking metrics exhibited significant intercepts (p < 0.05) in the OLS regression models, indicating substantial baseline phase times independent of cognitive skill metrics. However, the intercepts for velocity (p = 0.285), toe clearance (p = 0.63), and shoulder asymmetry (p = 0.535) were not significant. Working memory performance was significantly associated with velocity (B = 0.1769, p = 0.021) and stride length (B = 0.1582, p = 0.004). Mental rotations were significantly associated with shoulder asymmetry (B = 0.3349, p = 0.006).

**Table 11:** Ordinary least squares regression analysis examining the relationship between dual-task (DT) walking metrics and cognitive test scores.

Walking Metric	Predictor	B	SE	t	p	95% CI (Lower)	95% CI (Upper)	R²	Adj. R²
Velocity (DT)	Intercept	0.3043	0.274	1.11	0.285	−0.28	0.889	0.439	0.327
	Response Inhibition	−0.0038	0.006	−0.596	0.56	−0.017	0.01		
	Working Memory	0.1769	0.069	2.574	0.021*	0.03	0.323		
	Mental Rotations	0.0006	0.002	0.252	0.804	−0.004	0.005		
Stride Length (DT)	Intercept	0.4388	0.185	2.369	0.032*	0.044	0.833	0.589	0.507
	Response Inhibition	−0.0081	0.004	−1.906	0.076	−0.017	0.001		
	Working Memory	0.1582	0.046	3.409	0.004*	0.059	0.257		
	Mental Rotations	0.0026	0.001	1.727	0.105	−0.001	0.006		
Double Support (DT)	Intercept	0.4817	0.062	7.78	<0.001*	0.35	0.614	0.116	−0.06
	Response Inhibition	0.0008	0.001	0.527	0.606	−0.002	0.004		
	Working Memory	−0.0192	0.016	−1.239	0.234	−0.052	0.014		
	Mental Rotations	0	0.001	−0.068	0.947	−0.001	0.001		
Toe Clearance (DT)	Intercept	0.0052	0.011	0.492	0.63	−0.017	0.028	0.287	0.145
	Response Inhibition	−0.0001	0	−0.465	0.648	−0.001	0		
	Working Memory	0.0047	0.003	1.76	0.099	−0.001	0.01		
	Mental Rotations	0	0	0.41	0.688	0	0		
Hip ROM (DT)	Intercept	27.0852	7.458	3.631	0.002*	11.188	42.983	0.484	0.381
	Response Inhibition	0.29	0.172	0.169	0.868	−0.337	0.395		
	Working Memory	3.6951	1.87	1.976	0.067	−0.29	7.68		
	Mental Rotations	0.0393	0.06	0.652	0.524	−0.089	0.168		
Shoulder Asymmetry (DT)	Intercept	−8.1902	12.913	−0.634	0.535	−35.713	19.333	0.444	0.332
	Response Inhibition	−0.457	0.297	−1.538	0.145	−1.091	0.176		
	Working Memory	1.8923	3.237	0.585	0.568	−5.007	8.791		
	Mental Rotations	0.3349	0.105	3.204	0.006*	0.112	0.558		

The MANOVA results indicated that cognitive scores were collectively associated with the walking performance in the dual-task condition (Table 12). The overall model was significant (Wilks’ lambda = 0.0310, F(6, 10) = 52.0835, p < 0.0001). However, none of the individual cognitive scores (RI, WM, MR) reached significance (p > 0.05) in predicting the walking metrics under dual-task conditions.

**Table 12:** Multivariate analysis of variance (MANOVA) results assessing the impact of cognitive test scores on walking metrics under dual-task (DT) conditions.

Predictor	Test	Value	F Value	Num DF	Den DF	p-Value
**Intercept**	Wilks’ lambda	0.031	6	10	52.0835	< 0.001*
	Pillai’s trace	0.969	6	10	52.0835	< 0.001*
	Hotelling-Lawley trace	31.2501	6	10	52.0835	< 0.001*
	Roy’s greatest root	31.2501	6	10	52.0835	< 0.001*
**Response Inhibition**	Wilks’ lambda	0.5918	6	10	1.1497	0.4021
	Pillai’s trace	0.4082	6	10	1.1497	0.4021
	Hotelling-Lawley trace	0.6898	6	10	1.1497	0.4021
	Roy’s greatest root	0.6898	6	10	1.1497	0.4021
**Working Memory**	Wilks’ lambda	0.436	6	10	2.1561	0.1353
	Pillai’s trace	0.564	6	10	2.1561	0.1353
	Hotelling-Lawley trace	1.2937	6	10	2.1561	0.1353
	Roy’s greatest root	1.2937	6	10	2.1561	0.1353
**Mental Rotations**	Wilks’ lambda	0.4543	6	10	2.0022	0.1586
	Pillai’s trace	0.5457	6	10	2.0022	0.1586
	Hotelling-Lawley trace	1.2013	6	10	2.0022	0.1586
	Roy’s greatest root	1.2013	6	10	2.0022	0.1586

## Discussion

This study investigated the relationships between specific cognitive skills and gait performance in individuals with early-stage PD. Although global cognitive deficiency is observed in CI and dementia, certain cognitive skills may be more susceptible to decline depending on environmental factors and individual phenotype. In non-PD populations, Jayakody et al. [67] and Toots et al. [68] both reported gait velocity to have the strongest relationship with executive and visuospatial functions in comparison to other cognitive domains. In individuals with PD and mild CI, Amboni et al. [45] found visuospatial orientation to be strongly correlated to gait velocity. Recently, Piet et al. [69] found gait speed and step variability during DT walking to predict performance on executive function in older patients with PD. Consistent with this prior research, we found that motor-cognitive interference is evident even in high-functioning PD patients. Our results extend previous findings identifying which cognitive domains are most strongly associated with gait impairments. We report WM and MR abilities have strong correlations with locomotor behavior, especially under DT conditions and during TUG. Our observations reveal that in this PD population, specific cognitive domains are highly correlated to fine kinematic details such as velocity, stride length, hip and ankle ROM, and turning performance. All kinematic variables during ST gait were positively associated with WM. During normal walking, lower WM was associated with slower velocity, shorter stride length, reduced hip ROM, and poor toe clearance. These relationships were pronounced during DT, suggesting reduced neural compensation to the increased cognitive load. Participants with stronger WM are likely to have more intact or compensatory fronto-striatal function, enabling them to better maintain feedforward gait processes or devote additional attention to gait when needed. Phase times of TUG were negatively correlated with WM. Specifically, Sit-to-Stand, Turn Around, and Walk 2 phases were the strongest associations. WM is a complex task requiring activation of several cortical regions including frontal-associative and temporal regions with neostriatal reciprocal circuitry. WM also involves attention and goal-oriented behavior partially mediated by frontal eye-fields and subcortical visuomotor substrates [70]. The engagement of visuomotor neural circuits enables a mental image of WM problem solving tasks. Visuomotor dysfunction is common in PD and associated with cognitive [71] and gait [72,73] performance. The parallel processing necessary for healthy WM increases metabolic demand on fronto-striatal neural networks. Hence, the neural activation patterns in PD during WM tasks are heterogenous. Hyperactivation is observed with difficult WM tasks in cognitively normal PD patients while there is network hypoactivity in those with CI [30]. As such, individuals with PD that have poor WM may also struggle with locomotion, particularly under DT, because they cannot marshal the neural resources necessary to compensate for motor automaticity deficit. According to our data, WM has a strong correlation to locomotion in PD and may be a prime indicator of overall neural integrity and health.

The correlations of MR and gait kinematics were weak-to-moderate for ST and DT conditions. Specifically, SL and hip ROM were positively associated and approached clinical significance for fall risk [74,75]. Mental rotations and TUG were highly correlated with Turn Around and Walk 2 phases revealing strong negative associations. Turning around an object while walking challenges balance, limb placement, visuospatial awareness, and motor planning. Both visuospatial awareness and motor planning are components of MR and involve frontal, posterior, and subcortical structures [37]. Hence, in addition to motor circuitry dysfunction [25,76], visuospatial substrates of MR may be factors in the etiology of turning difficulties observed in PD.

In this study, RI showed weak correlation to gait kinematics and TUG performance. Although RI was not as strongly correlated to locomotion as MR or WM, the findings may be a subtle expression of global cognitive deficiency often observed with neurodegenerative disease and aging. Various RI tasks may provide different neural substrate challenges based on task context such as impulse control, error correction, and conflict resolution [32,77]. The Stroop color-word task was selected to challenge frontal attentional and conflict resolution as opposed to a “go-no-go” task which has an inherently stronger motor response component. Using a RI task with minimal motor demand decreased the risk of individual motor dysfunction influencing RI performance; thus, improving the specificity of cognitive assessment for gait correlation analysis. Manza et al. [78] reported RI deficiency in older individuals with more severe PD burden compared to those newly diagnosed. Collectively, our study participants were in the early diagnostic stage, highly functional with low UPDRS motor scores, and healthy MoCA screenings. As such, it is possible that our cohort did not reach a clinically deficient threshold of RI to reveal a stronger relationship.

The interaction of motor and cognitive deficiency, referred to as the motoric-cognitive risk syndrome [79], begins with subtle signs and progresses during aging and/or neurodegenerative processes. The initial sign of motoric-cognitive risk syndrome is bradykinetic gait often with subjective cognitive complaints. Purks et al. [80] recently reported that cognition is a common concern in PD with >30% of patients reporting a cognitive symptom as the most bothersome. These subjective symptoms are often missed by cognitive screening tools, while more sensitive neuropsychological batteries are not routinely administered in early stages of PD. Typically, PD has been a clinical diagnosis based on overt signs of rigidity, tremor, and bradykinesia along with physiological symptoms (*i.e.*, hyposmia, constipation). Patients presenting with uncertain or atypical clinical signs may receive special imaging (*i.e.*, DaTscan) or, more recently, the newly developed alpha-synuclein seed analysis [81]. These advanced procedures improve the specificity and sensitivity of diagnosis but do not provide means to measure physical function nor predict future impairments. To this end, gait assessment has been proposed as a vital biomarker for detection and measure of progression in PD [20,21]. Moreover, early gait analysis may be a predictor of cognitive impairments during aging and/or disease advancement [69,82]. Thus, given the consistent data supporting the use of gait as an assessment tool in PD, we suggest gait is a vital component of the neurological evaluation in all stages of disease. New gait analysis technologies offer a high yield, low risk tool feasible for clinical use in the neurological setting [55,83]. Further, global and specific cognitive assessments in the early diagnostic stage may help reveal impairment risk. Due to the inherent neuropathological processes, PD has a strong predictive incidence of motor-cognitive risk syndrome [79] suggesting detection of both gait and cognitive deficiencies are critical for comprehensive patient care during all phases of disease management. Our findings suggest these deficiencies may have specific relationships that may influence clinician strategies for management and rehabilitation.

## Limitations

We acknowledge several limitations of this study. The small sample (68% female) limits statistical power and may not be generalizable. A larger sample may have revealed significant and/or stronger correlations between cognitive domains (*i.e.,* RI) and gait parameters. We also acknowledge the lack of a control group which may limit internal validity. However, the evidence is consistent that motor and cognitive deficiencies are accelerated in PD compared to healthy aging controls. Our findings suggest there may be specific cognitive domain-gait parameter interactions that likely follow this same pattern. Participants were enrolled from support groups and a private practice, and as such, may have been highly motivated, altruistic, and interested in learning about their condition [84]. Despite these limitations, our results are consistent with previous research and provide additional considerations on the presence of heterogenous cognitive domains with correlation to gait dysfunction in early PD. Our study participants were (a) highly educated, (b) relatively newly diagnosed, in early Hoehn and Yahr stage, with low UPDRS scores, (c) did not meet screening criteria for cognitive impairment, (d) did not report depression, and (e) maintained their medication schedule. Nevertheless, we report distinct correlations between gait and cognitive function in these individuals. Still, given the limitations stated above, these findings are preliminary and should be interpreted with caution. Future researchers may wish to compare different types of DT (e.g., cognitive vs. motor dual tasks, different cognitive domains) to observe which best reveal gait vulnerabilities. Additionally, intervention studies could explore whether targeted therapies such as DT training and/or customized cognitive rehabilitation (*i.e.,* WM tasks) can enhance both cognition and gait, thereby potentially slowing the progression of the motoric-cognitive risk syndrome in PD. Future investigations may also wish to measure motor-cognitive correlations longitudinally in a more diverse, controlled sample.

## Conclusion

Our findings provide insights into the motor-cognitive interactions in a cohort of individuals with PD. Motor and cognitive deficits may progress concurrently over the course of disease progression. Specifically, our data suggests WM and MR are strong cognitive correlates to locomotor function in PD, particularly to velocity, stride length, and hip ROM. Understanding these relationships can potentially help researchers and clinicians develop effective strategies focused on maintaining mobility and cognitive function in individuals with PD. For example, specific DT interventions using WM tasks and MR/visuospatial challenges may enhance gait training and rehabilitative sessions. Further, these findings may encourage new study of neural substrate pathology, mapping, and PD topology to help further our understanding of PD pathophysiology.
